# Sex-Based Differences in Clinical Characteristics and Outcomes among Patients with Peripheral Artery Disease: A Retrospective Analysis

**DOI:** 10.3390/jcm12155094

**Published:** 2023-08-03

**Authors:** Giuseppe De Matteis, Federico Biscetti, Davide Antonio Della Polla, Amato Serra, Maria Livia Burzo, Mariella Fuorlo, Maria Anna Nicolazzi, Angela Novelli, Angelo Santoliquido, Giovanni Gambassi, Antonio Gasbarrini, Andrea Flex, Francesco Franceschi, Marcello Covino

**Affiliations:** 1 Department of Internal Medicine, Fondazione Policlinico Universitario A. Gemelli, IRCCS, 00168 Rome, Italy; giuseppe.dematteis@policlinicogemelli.it (G.D.M.);; 2 Cardiovascular Internal Medicine, Fondazione Policlinico Universitario A. Gemelli, IRCCS, 00168 Rome, Italy; 3 Emergency Department, Fondazione Policlinico Universitario A. Gemelli, IRCCS, 00168 Rome, Italy; 4 Department of Internal Medicine, Ospedale Santo Spirito in Sassia, 00193 Rome, Italy; 5 Faculty of Medicine and Surgery, Rome Campus, Università Cattolica del Sacro Cuore, 20123 Rome, Italy

**Keywords:** peripheral artery disease, sex, limb amputations, revascularization, MACE, MALE

## Abstract

Peripheral arterial disease (PAD) is a prevalent medical condition associated with high mortality and morbidity rates. Despite the high clinical burden, sex-based differences among PAD patients are not well defined yet, in contrast to other atherosclerotic diseases. This study aimed to describe sex-based differences in clinical characteristics and outcomes among hospitalized patients affected by PAD. This was a retrospective study evaluating all patients with a diagnosis of PAD admitted to the Emergency Department from 1 December 2013 to 31 December 2021. The primary endpoint of the study was the difference between male and female PAD patients in cumulative occurrence of Major Adverse Cardiovascular Events (MACEs) and Major Adverse Limb Events. A total of 1640 patients were enrolled. Among them, 1103 (67.3%) were males while females were significantly older (median age of 75 years vs. 71 years; *p* =< 0.001). Females underwent more angioplasty treatments for revascularization than men (29.8% vs. 25.6%; *p* = 0.04); males were treated with more amputations (19.9% vs. 15.3%; *p* = 0.012). A trend toward more MALEs and MACEs reported in the male group did not reach statistical significance (OR 1.27 [0.99–1.64]; *p* = 0.059) (OR 0.75 [0.50–1.11]; *p* = 0.153). However, despite lower extremity PAD severity seeming similar between the two sexes, among these patients males had a higher probability of undergoing lower limb amputations, of cardiovascular death and of myocardial infarction. Among hospitalized patients affected by PAD, even if there was not a sex-based significant difference in the incidence of MALEs and MACEs, adverse clinical outcomes were more common in males.

## 1. Introduction

Peripheral arterial disease (PAD) is a progressive atherosclerotic disorder characterized by partial or complete occlusion of at least one lower extremity artery [1]. The prevalence of PAD is increasing [2] and more than 200 million adults worldwide are suffering from PAD, almost three-quarters of them coming from low- and middle-income countries [3]. However, the real incidence and prevalence are underestimated because PAD remains frequently underdiagnosed due to a lack of physician and patient awareness [4]. Clinical manifestations of PAD are extremely heterogeneous depending on both the localization and severity of arterial stenosis or occlusion. Indeed, symptoms can range from mild lower extremity pain to intermittent claudication and chronic limb-threatening ischaemia (CLTI) in the most severe cases [5]. Many trials have been conducted trying to understand what factors predispose CLTI patients to complications and trying to find possible biomarkers of disease severity and progression [6,7].

Several studies analysed sex-based differences among patients affected by atherosclerotic diseases [8]. Current evidence suggests that stroke incidence is higher in women than men among those aged <30 years, lower in women in mid-life and similar in those aged ≥80 years [9], women tending to have a worse prognosis [10]. Similarly, men showed a higher prevalence of obstructive coronary artery disease (CAD) compared to women, while non-obstructive CAD and ischaemia with non-obstructive coronary arteries were more commonly described in women [11].

Likewise, the interaction of sex-based factors may affect PAD patients [12]. Women affected by PAD seem to be more commonly asymptomatic and report atypical symptoms more often than men, with lower rates of claudication intermittens (CI), probably contributing to both decreased and delayed PAD detection in female patients [13]. Moreover, a meta-analysis revealed that acute thrombosis is more common in men than women, whereas plaque rupture is equally common across all age groups of men [14]. Furthermore, women with PAD, compared to men, have poorer quality of life because they are older, with multiple vessel involvement and more advanced disease [15]. In addition, studies demonstrated that women with symptomatic PAD were less likely to receive goal-directed medical therapy and showed a lower rate of open surgical revascularizations compared with men [16], while data about post-revascularization outcomes showed mixed results between the two sexes, with a higher risk of developing periprocedural complications after percutaneous revascularizations in female patients [17].

To date, despite this evidence, knowledge about sex-based differences in clinical characteristics, and cardiovascular and limb outcomes among patients with PAD is still limited [18].

This study aims to describe the differences in clinical characteristics and outcomes between patients of different sexes suffering from PAD and admitted to the Emergency Department (ED). The aim is to define the sex-based characteristics of the disease and to enable the design of a personalized diagnosis and treatment pathway.

## 2. Materials and Methods

### 2.1. Study Population

This was a single-centre, retrospective study conducted at the Fondazione Policlinico A. Gemelli IRCCS (Rome, Italy), an academic medical centre where the ED has an average annual attendance of approximately 80,000 adult patients.

We included in the study sample patients who received a definitive diagnosis of PAD who were admitted to the ED and subsequently hospitalized from 1 December 2013 to 31 December 2021.

The adjudication criteria included both an admission diagnosis of PAD and the presence of PAD in the hospital discharge record. Diagnosis at hospital discharge was based on the codes according to the “International Classification of Disease, tenth revision” (ICD-10 CM). 

The definition of PAD was based on at least one measure of the ankle–brachial index (ABI) < 0.9 and the evidence of atherosclerosis at the ultrasonographic evaluation [19] of the lower limb arteries in the patient’s past clinical history. We included in the study only patients who received a diagnosis of PAD more than one year before the ED presentation.

Patients under 18 years of age and those diagnosed with COVID-19 at ED admission were excluded from the analysis. 

### 2.2. Study Variables

We obtained data through consultation of electronic medical records, and each patient’s health record was used to collect demographic and clinical characteristics, data regarding ED presentation, and events occurring during the hospital stay, including diagnostic and therapeutic procedures, and the outcome at discharge. 

Clinical records were reviewed to assess comorbidities based on the patient’s history and hospital discharge diagnosis. We evaluated the following information:

- Demographic data: age and sex.
- Clinical presentation at the ED admission including dyspnoea, fever, pain (defined as any degree and type of pain in the lower extremities), chest pain and neurological symptoms.
- Peripheral artery disease grade at ED presentation, according to Rutherford classification.
- Coexistent acute infections diagnosed at ED admission, categorized as bloodstream infections, lower limb and other infections not included in the previous categories.
- Laboratory findings including blood creatinine, eGFR (estimated Glomerular Filtration Rate calculated according to the CKD-EPI equation), urea, glucose, glycated haemoglobin (HbA1c), C-reactive protein (CRP), total cholesterol, high-density lipoprotein (HDL), low-density lipoprotein (LDL), triglycerides, haemoglobin (Hb) and platelets (PLTs).
- Clinical history and comorbidities: CAD, congestive heart failure (CHF), cerebral vascular disease—previous stroke (CVD), type 2 diabetes mellitus (T2DM), chronic obstructive pulmonary disease (COPD), chronic kidney disease (CKD) and dementia. Overall comorbidity severity was assessed by Charlson Comorbidity Index (CCI) [20].

### 2.3. Study Endpoints

The primary endpoint of the study was the difference between male and female PAD patients in cumulative occurrence of Major Acute Cardiovascular Events (MACEs), defined as nonfatal stroke, nonfatal myocardial infarction and cardiovascular death, and in cumulative occurrence of Major Acute Limb Events (MALEs), defined as limb acute ischaemia, need for urgent revascularization or amputation (any kind of amputation). 

Secondary endpoints were all-cause in-hospital deaths and length of hospital stay (LOS). 

### 2.4. Statistical Analysis

Categorical variables are presented as numbers and percentages. Continuous normally distributed variables are presented as mean ± standard deviation, non-normally distributed data are presented as median (inter-quartile range), and binary or ordinal variables are presented as absolute frequency (%). The LOS was calculated from the time of ED admission to discharge or death. Parametric variables were compared by the Mann–Whitney U test, whereas categorical variables were compared by the chi-square test (with Fisher test if indicated). 

Significant variables at univariate analysis were entered into a multivariate logistic regression model to identify independent predictors for the evaluated endpoints. Gender was forced in all the analyses. To avoid overfitting and overestimation of the parameters, the variables with high collinearity were excluded from the multivariate models. If possible categorical variables were preferred to continuous ones. The single items composing cumulative variables (i.e., Charlson index) were excluded from the model to avoid redundancy. Finally, laboratory values were excluded from the analysis for the high collinearity with the categorical descriptors. In multivariate analyses of MALE events, the Rutherford classification established at the time of admission to the ED was not included among the predictors, as it overlapped by definition with the outcome analysed. The results of the logistic regression analysis are reported as Odds Ratio (OR) (95% confidence interval). 

A two-sided *p*-value of 0.05 or less was considered significant in all the analyses. All data were analysed by SPSS v26^®^ (IBM, Armonk, NY, USA).

## 3. Results

### 3.1. Study Cohort and Baseline Characteristics

Overall, 1640 patients with a diagnosis of PAD were evaluated in the ED in the study period [Table 1]. 

Among them, 1103 (67.3%) were male. Females affected by PAD admitted to the ED were significantly older, with a median age of 75 years (69–84) vs. 71 years (63–80) in the male group. The most common symptom on ED admission was pain, reported by about half of the patients, without significant gender differences (42% vs. 44.9%; *p* = 0.144). Most patients (54.1%) were categorized as Rutherford Grade III-6 at ED admission, and no statistically significant differences were found in Rutherford’s classification between males and females. However, the male group showed a more frequent history of limb amputation (17.9% vs. 12.7%; *p* = 0.004).

History of CAD (45.1% vs. 37.6%) and diabetes (79.8% vs. 74.5%) were more prevalent in men. No relevant gender differences were found among other comorbidities examined in the study. 

Furthermore, the male group showed a higher average creatinine level, lower eGFR, lower platelet count and lower HDL level, while higher values of total cholesterol and triglycerides were reported in the female group. No differences were observed in terms of HDL- or LDL-cholesterol, or regarding HbA1c levels.

The female group underwent more angioplasty treatments for revascularization compared with men (29.8% vs. 25.6%). Conversely, a significantly higher percentage of the male group had been treated with amputation (19.9% vs. 15.3%). The percentages of acute limb ischaemia (ALI) and urgent revascularizations were similar between the two genders. Lastly, no statistically significant difference in all-cause mortality or LOS was found between males and females.

### 3.2. MALE Composite Outcome

MALE events were reported in 660 patients [Table 2]. The events occurred in 206 (31.2% of total events) females and 454 (68.8% of total events) males. Although MALE events were slightly more common in the male group there was not a statistically significant gender-related difference (OR 1.27 [0.99–1.64]; *p* = 0.059). The presence of pain at ED admission was significantly predictive of subsequent MALE (OR 3.65 [2.85–4.70]; *p* < 0.001). Similarly, an advanced Rutherford stage at ED presentation was prognostic for MALEs. Indeed, patients categorized as Rutherford Grade III-6, indicative of more severe disease, experienced more commonly a MALE (59.6% vs. 36.6%; *p* < 0.001).

Furthermore, higher blood levels of HbA1c and CRP at ED admission were reported in patients who experienced a MALE. 

History of previous amputation was also significantly associated with MALEs (18.8% vs. 14.4%; *p* = 0.011). Predictably, lower-limb infections were statistically significantly associated with MALEs (OR 18.9 [9.72–36.87]; *p* = 0.001). Furthermore, a coexistent acute bloodstream infection was more commonly reported in the MALE group (10.3% vs. 6.3%; *p* = 0.004). Among comorbidities, patients who experienced MALEs showed a higher diabetes rate (80.5% vs. 76.4%; *p* = 0.008).

Finally, LOS was higher in patients with MALEs (19.3 vs. 13.1; *p* < 0.001), while no statistically significant differences in mortality were observed between the two groups.

### 3.3. MACE Composite Outcome

Overall, 123 patients reported a MACE [Table 3]. MACEs were more frequent in men (67.8%; *p* = 0.020) without a statistically significant gender-related difference (OR 0.75 [0.50–1.11]; *p* = 0.153). Furthermore, as expected, MACEs were most frequently observed in patients hospitalized for chest pain (OR 3.46 [1.82–6.56]; *p* < 0.001) and neurological symptoms (OR 2.96 [1.49–5.89]; *p* < 0.001).

Higher creatinine levels with lower eGFR and higher triglyceride blood levels were more associated with MACE. In our sample, no statistically significant correlation was found among total, HDL- and LDL-cholesterol levels, and MACE risk, although higher values were reported in the MACE group compared with controls.

Patients who experienced MACE events had more commonly a history of CAD (59.3% vs. 41.3%; *p* < 0.001), CHF (34.1% vs. 26.7%; *p* = 0.017) or CVD (15.4% vs. 8.6%; *p* = 0.021).

**Figure 1:**
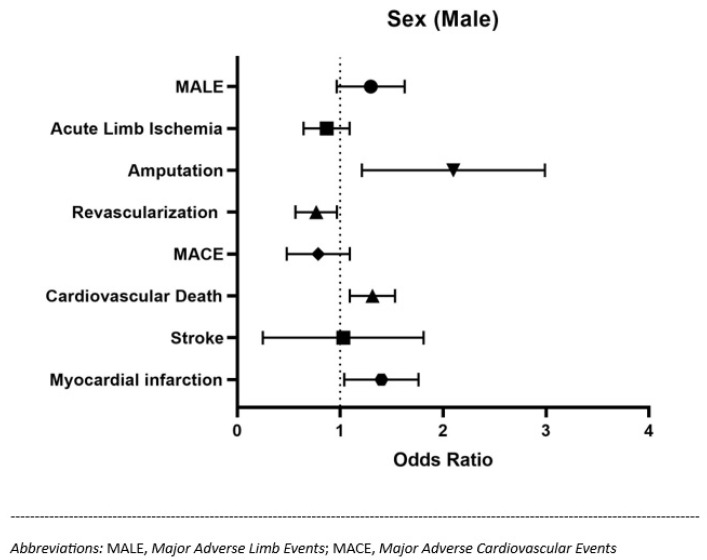
Forest Plot showing the odds ratio and 95% confidence intervals for MACEs, MALEs and relevant clinical outcomes in the male population of the sample.

Furthermore, we found a longer hospital stay (18.8 vs. 15.7; *p* = 0.011) and a higher rate of all-cause in-hospital death (37.4% vs. 5.3%; *p* < 0.001) in patients with MACEs. However, despite no statistically significant differences in MACEs and MALEs, we found relevant sex-based differences in our study sample (Table 1). Male sex in patients affected by PAD was associated with a higher probability of undergoing lower limb amputations, and a higher rate of cardiovascular death and myocardial infarction (Figure 1). Conversely, women with PAD were more likely to be treated with revascularization procedures. No sex-related differences were found in terms of acute limb ischaemia.

## 4. Discussion

The prevalence of PAD is increasing worldwide, especially in low- and middle-income countries [21], and it remains frequently underdiagnosed due to a lack of physician and patient awareness [4]. The 5-year follow-up of asymptomatic PAD patients shows a 7% chance of developing CI and cardiovascular mortality is higher in these patients [22]. Consequently, PAD is both a common and critical medical condition that requires prompt diagnosis and management in ED [1,4]. Moreover, even if patients with PAD have the highest rates of cardiovascular death and major cardiovascular events among patients with atherosclerotic disease [15], sex-based differences among PAD patients are not well defined yet, in contrast to CAD and CVD.

Thus, ascertaining sex-based disparities continues to be a challenge in PAD research, as women are still underrepresented in clinical and treatment trials, which may be partially related to the differences both in the nature and timing of clinical manifestations of atherosclerosis [4,23,24,25]. Indeed, although call-to-action initiatives have made progress, only around one-third of women currently participate in clinical trials and this results in small sample sizes of females and low statistical power for identifying remarkable sex-related outcomes [23]. As a result, not only is it difficult for clinical studies to identify sex-based differences but it also leads to reduced adequate care for women, which ultimately results in years of life lost with a disability. 

In our population, we did not find a statistically significant difference in the incidence of composite outcomes, such as MALEs and MACEs, between the two sexes. Nevertheless, even if a *p*-value of 0.059 is not significant, the sex difference regarding MALEs shows a strong trend towards more events for the male population. Notably, our study showed that males had a higher risk of amputation. To date, there is insufficient evidence or data from surveys about sex-related differences in cardiovascular event rates in PAD populations [24]. Similarly to our report, a prospective trial conducted in a tertiary centre in France showed no statistically significant differences between the two sexes in MALEs and MACEs among hospitalized patients with PAD at 1-year follow-up, despite women smoking less, having less CAD and having a trend for fewer MACEs and increased MALEs compared with men [25]. Sigvant et al. showed that, despite no sex differences in cardiovascular death risk in PAD patients, male patients had a one-third higher risk of all-cause mortality over a follow-up period of at least one year [26]. According to a propensity score-matched analysis of inpatients in Germany, male PAD patients were found to be at an increased mortality risk during a 4-year follow-up period [27]. Conversely, two cohort studies discovered no sex-related differences in cardiovascular mortality risk, including PAD patients [28]. Likewise, in the Examining Use of Ticagrelor in Peripheral Artery Disease (EUCLID) trial, including a higher proportion of men participants, women affected by PAD were at lower risk for MACE and all-cause mortality, while the rate of MALE was similar between genders over an average follow-up of 30 months [29].

Women were significantly older than men in our sample; in interpreting the data, it should be considered that age also increases the risk of developing PAD-related adverse events. Two other studies including a sample of women affected by PAD older than men showed no differences in the incidence of MALEs and MACEs between the two genders over a follow-up period after percutaneous revascularization [30,31]. Furthermore, we found that PAD patients who developed a MACE presented adverse clinical outcomes, including not only an increased rate of all-cause in-hospital death but also an increased length of hospital stay, as previously reported in the literature [32,33]. Interestingly, increased triglyceride levels are associated with an increased risk of MACE in the study population. This seems to be a further indication that, while LDL-cholesterol represents one of the general major cardiovascular risk factors, triglycerides and, in particular, the ratio of triglycerides to HDL-cholesterol, are a promising biomarker of vascular risk in patients with PAD [34].

In addition, our analysis showed that male patients affected by PAD were treated more frequently with limb amputation procedures, despite PAD severity seeming to be similar between the two sexes. Conversely, women underwent more revascularization procedures, such as percutaneous angioplasty and stent placement. Indeed, this could be relevant from a clinical perspective, as PAD in males may represent a disease with foreseeable long-term disability effects. Furthermore, this result is in agreement with a study conducted on the Nationwide Inpatient Sample which showed that, despite the yearly increase in endovascular procedures for both sexes, women were more likely to undergo endovascular than surgical revascularizations compared to men [35].

Inflammation certainly plays a role in the development and worsening of atherosclerosis, which underlies PAD [6,7,36,37]. In this scenario, indirect mechanisms could justify the differences between the two sexes [16]. On the one hand, sex-based PAD differences may be due to hormonal and physiological differences between men and women. Indeed, oestrogen has been shown to have protective effects on the cardiovascular system, potentially lowering the risk of PAD in women [38]. As is known, oestrogens modulate endothelial functions in women through antioxidant and anti-inflammatory effects. As a consequence, changes in sex hormones with the menopause lead to endothelial dysfunction which is an initial step in the etiopathogenesis of atherosclerosis and PAD. Endothelial dysfunction occurs after menopause at least a decade later than in men with subsequent acceleration to similar impairment to that seen in males [15]. On the other hand, women may have smaller arteries and a higher percentage of body fat, which could influence PAD development and progression [39]. In our population, the different exposure to risk factors in male patients, combined with the relative protection of oestrogen in the premenopausal period for women, could explain the increased severity of PAD that led to amputations in men.

In addition, social and behavioural factors may also influence sex-based PAD differences. A previous report showed a relationship between environmental pollution, lower socioeconomic status and lower educational level with a higher incidence of PAD [15]. Moreover, men seem to underestimate PAD symptoms, leading to delayed diagnosis and treatment [40]. To explain this, one hypothesis was that male patients tend to seek medical attention late when the disease is advanced to the point that endovascular treatments cannot be undertaken. A second possible hypothesis could be that women develop serious cardiovascular disease at least 10 years later than men [27,35]. Consequently, these factors could be related to a more severe form of PAD in men at diagnosis. This would result in a need for more invasive treatment than in women at disease onset.

Furthermore, our study showed that male patients with PAD were more frequently affected by diabetes and had lower HDL-cholesterol blood levels than women. This is in line with previous data showing that men had a higher prevalence of risk factors for PAD, such as hypertension and diabetes, and were more likely to smoke compared to women [41].

Thus, a call to action by the American Heart Association was issued in response to inconsistent results on outcomes by sex, highlighting the need for more reliable data and its implications [42]. Indeed, PAD remains plagued by sex discrepancies. Furthermore, the data from this study add some decision-making and predictive elements for clinicians dealing with PAD and its complications in an emergency setting.

Further scientific evidence is needed to identify missed opportunities. Indeed, because of the complex network of factors involved in determining the risk for PAD, it could be important to have a systemic approach exploring how sex-specific biological factors, gender-related behavioural determinants, including lifestyle factors (related to smoking, drinking, weight and physical activity), and social factors can interact to influence sex-based differences in these patients. We strongly encourage future researchers on PAD to routinely include a fair percentage of both sexes.

## 5. Limitations

Our research had some limitations. Firstly, this is a single-centre study and the enrolled population could not be representative of all PAD patients. However, our hospital has a dedicated team of PAD specialists that could make the analysis very accurate. Secondly, being a retrospective analysis, all data were extracted from electronic records, and our classification of cases and diagnosis was based on the information available in hospital records. Thirdly, the sample size of men is twice that of women, which may have influenced the results. Furthermore, due to the study design it was not possible to include social and behavioural factors, such as socioeconomic status, education level, access to healthcare, physical activity levels and diet, into the data analysis, so we cannot rule out that they may have influenced the observed results. Lastly, it was not possible to collect the follow-up data of these patients after hospital discharge which may lead to under estimating MACEs and MALEs.

## 6. Conclusions

Even though there was no statistically significant difference in the incidence of MALEs and MACEs among hospitalized PAD patients, males had more adverse clinical outcomes. Even though the severity of lower extremity PAD appears to be similar between the sexes, males had a higher risk of lower limb amputations, cardiovascular mortality and myocardial infarction. Women with PAD, on the other hand, were more likely to undergo revascularization. These disparities between invasive and non-invasive treatment may indicate major differences in pathophysiology, diagnostic delay and risk factor treatment. More research is needed in order to investigate the potential for better characterization and tailored management of gender differences in PAD patients.

## Figures and Tables

**Table 1:** Clinical characteristics of the enrolled patients at ED admission.

	All Cases *N* 1640	Male *N* 1103	Female *N* 537	*p* Value
Age	72 [64–81]	71 [63–80]	75 [69–84]	**<0.001**
Clinical presentation				
Fever	425 (25.9%)	301 (27.3%)	124 (23.1%)	**0.038**
Pain	704 (42.9%)	463 (42%)	241 (44.9%)	0.144
Chest pain	107 (6.5%)	82 (7.4%)	25 (23.4%)	**0.019**
Dyspnoea	184 (11.2%)	136 (12.3%)	48 (8.9%)	**0.024**
Neurological symptoms	67 (4.1%)	48 (4.4%)	19 (3.5%)	0.261
PAD grade (according to Rutherford classification)				
Rutherford Grade I-3	101 (6.2%)	73 (6.6%)	28 (5.2%)	
Rutherford Grade II-4	171 (10.4%)	106 (9.6%)	65 (12.1%)	
Rutherford Grade III-5	161 (9.8%)	107 (9.7%)	54 (10.1%)	0.158
Rutherford Grade III-6	888 (54.1%)	595 (53.9%)	293 (54.6%)	
Previous amputation	265 (16.2%)	197 (17.9%)	68 (12.7%)	**0.004**
Presence of infections				
Bloodstream infection	130 (7.9%)	92 (8.3%)	38 (7.1%)	0.215
Lover limb infections	888 (54.1%)	595 (53.9%)	293 (54.6%)	0.813
Other infections	1043 (63.6%)	710 (64.4%)	333 (62.0%)	0.352
Laboratory findings				
Creatinine (mg/dL)	1.95 [0.83–2.07]	2.1 [0.86–2.32]	1.64 [0.74–1.71]	**<0.001**
eGFR (ml/min)	54.3 [28.5–84.2]	50.8 [29.4–76.7]	55.6 [28.0–88.7]	**0.024**
Urea (mg/dL)	33.2 [18–40]	33.6 [18–42]	32.4 [18–39]	0.186
Glucose (mg/dL)	173 [107–207]	172 [108–207]	174 [104–208]	0.443
HbA1c (mmol)	61 [45–72]	62 [44–74]	61 [45–71]	0.848
C-reactive protein (mg/L)	95 [19–148]	99 [25–152]	88 [14–134]	0.160
Total cholesterol (mg/dL)	129 [102–150]	124 [97–147]	138 [110–161]	**<0.001**
HDL (mg/dL)	32 [24–38]	30 [23–36]	36 [27–43]	**<0.001**
LDL (mg/dL)	75 [53–90]	72 [48–85]	74 [53–90]	0.077
Triglycerides (mg/dL)	128 [86–154]	122 [83–145]	139 [93–173]	**<0.001**
Hb (gr/dL)	11.3 [9.8–12.9]	11.53 [9.8–13.2]	11 [9.7–12.4]	**<0.001**
PLTs (×10^9^/L)	302 [204–369]	292 [196–357]	323 [218–400]	**0.001**
Comorbidities				
CCI	4 [3–6]	4 [3–6]	4 [2–5]	**0.020**
CAD	699 (42.6%)	497 (45.1%)	202 (37.6%)	**0.002**
Congestive heart failure	447 (27.3%)	314 (28.5%)	133 (24.8%)	0.064
Cerebrovascular disease	149 (9.1%)	98 (8.9%)	51 (9.5%)	0.374
Dementia	109 (6.6%)	67 (6.1%)	42 (7.8%)	0.111
COPD	196 (12%)	138 (12.5%)	58 (10.8%)	0.179
Diabetes	1280 (78%)	880 (79.8%)	400 (74.5%)	**0.009**
Chronic kidney disease	565 (34.5%)	391 (35.4%)	174 (32.4%)	0.122
Outcomes				
Death (all causes)	127 (7.7%)	79 (7.2%)	48 (8.9%)	0.123
Length of stay (LOS)	16 [7–20]	16 [7–21]	15 [7–19]	0.148
Acute limb ischaemia (ALI)	288 (17.6%)	187 (17.0%)	101 (18.8%)	0.357
Overall revascularizations	447 (27.3%)	286 (25.9%)	161 (30.1%)	**0.048**
Angioplasty	442 (27.0%)	282 (25.6%)	160 (29.8%)	**0.040**
Stenting	23 (1.4%)	14 (1.3%)	9 (1.7%)	0.325
Urgent revascularizations	267 (16.3%)	168 (15.2%)	99 (18.4%)	0.058
Amputations	302 (18.4%)	220 (19.9%)	82 (15.3%)	**0.012**
MALE cumulative events	660 (40.2%)	454 (41.2%)	206 (38.4%)	0.151
Myocardial infarction	60 (3.7%)	40 (3.6%)	20 (3.7%)	0.510
Stroke	28 (1.7%)	18 (1.6%)	10 (1.9%)	0.438
MACE cumulative events	123 (7.5%)	78 (7.1%)	45 (8.4%)	0.199

Abbreviations: HbA1c, Glycated haemoglobin; HDL, high-density lipoprotein; LDL, low-density lipoprotein; Hb, haemoglobin; PLT, platelets; PAD, peripheral artery disease; MALE, Major Adverse Limb Event; MACE, Major Adverse Cardiovascular Event; CCI, Charlson Comorbidity Index; CAD, coronary artery disease; COPD, chronic obstructive pulmonary disease.

**Table 2:** Univariate and multivariate analysis of factors associated with cumulative MALE events (Major Adverse Limb Events, including acute ischaemia, revascularization and amputation).

	Controls N 980	MALE Cumulative Events N 660	Unadjusted *p* Value	Adjusted Odds for MALE [95% Confidence Interval]	Multivariate *p* Value
Age	72 [65–81]	71 [64–80]	**0.030**	0.99 [0.98–1.01]	0.484
Sex (male)	649 (66.2%)	454 (68.8%)	0.151	1.27 [0.99–1.64]	0.059
Clinical presentation					
Fever	265 (62.4%)	160 (37.6%)	0.110		
Pain (any)	333 (47.3%)	371 (52.7%)	**<0.001**	3.65 [2.85–4.70]	**<0.001**
Chest pain	91 (9.3%)	16 (2.4%)	**<0.001**	0.25 [0.13–0.46]	**0.003**
Dyspnoea	166 (16.9%)	18 (2.7%)	**<0.001**	0.28 [0.16–0.49]	**0.006**
Neurological symptoms	45 (4.6%)	22 (3.3%)	**0.047**		
Laboratory findings					
Creatinine (mg/dL)	1.86 [0.84–2.02]	2.08 [0.82–2.2]	0.770		
eGFR	54.4 [29.3–82.3]	53.8 [26.5–88.4]	0.977		
Urea (mg/dL)	33.6 [18–41]	32 [18–39]	0.610		
Glucose (mg/dL)	174 [107–202]	171 [107–215]	0.780		
HbA1C (mmol)	57 [44–69]	66 [51–78]	**<0.001**		
C-reactive protein (mg/L)	87 [16–128]	107 [27–161]	**<0.001**		
Total cholesterol (mg/dL)	133 [104–157]	123 [99–145]	**<0.001**		
HDL (mg/dL)	32 [25–39]	33 [23–37]	0.140		
LDL (mg/dL)	73 [50–88]	73 [48–85]	0.399		
Triglycerides (mg/dL)	131 [87–158]	123 [86–151]	0.315		
Hb (gr/dL)	11.4 [9.8–13]	11.2 [9.7–12.9]	0.054		
PLT (×10^9^/L)	284 [194–344]	328 [230–404]	**<0.001**		
PAD grade (according to Rutherford classification)					
Rutherford Grade I-3	67 (6.8%)	34 (5.2%)			
Rutherford Grade II-4	125 (12.8%)	46 (7.0%)			
Rutherford Grade III-5	123 (12.6%)	38 (5.8%)	**<0.001**		
Rutherford Grade III-6	359 (36.6%)	529 (59.6%)			
Previous amputation	141 (14.4%)	124 (18.8%)	**0.011**	0.91 [0.67–1.25]	0.574
Presence of infections					
Bloodstream infection	62 (6.3%)	68 (10.3%)	**0.004**	1.75 [1.11–2.76]	**0.016**
Lower limb infections	359 (36.6%)	529 (80.2%)	**<0.001**	18.9 [9.72–36.87]	**0.001**
Other infections	503 (51.3%)	540 (81.8%)	**<0.001**	0.27 [0.13–0.55]	**<0.001**
Comorbidities					
CCI	4 [2–5]	4 [3–6]	**0.003**	1.03 [0.98–1.08]	0.267
CAD	412 (42%)	287 (43.5%)	0.576		
Congestive heart failure	282 (28.8%)	165 (25%)	0.101		
Cerebrovascular disease	99 (10.1%)	50 (7.6%)	**0.015**		
Dementia	70 (7.1%)	39 (5.9%)	0.360		
COPD	124 (12.7%)	72 (10.9%)	0.161		
Diabetes	749 (76.4%)	531 (80.5%)	**0.008**		
Chronic kidney disease	324 (57.3%)	241 (42.7%)	**0.015**		
Outcomes					
Length of stay (LOS)	13.1 [7.1–16.5]	19.3 [8.5–25.5]	**<0.001**		
Death (all causes)	76 (7.8%)	51 (7.7%)	0.075		

Abbreviations: HbA1c, glycated haemoglobin; HDL, high-density lipoprotein; LDL, low-density lipoprotein; Hb, haemoglobin; PLT, platelets; PAD, peripheral artery disease; CCI, Charlson Comorbidity Index; CAD, coronary artery disease; COPD, chronic obstructive pulmonary disease. Rutherford’s classification was excluded from the model to avoid redundancy and overfitting.

**Table 3:** Univariate and multivariate analysis of factors associated with cumulative MACE events (Major Acute Cardiovascular Events including acute myocardial ischaemia, stroke and cardiovascular death).

	Controls N 1517	MACE Cumulative Events N 123	Unadjusted *p* Value	Adjusted Odds for MACE [95% Confidence Interval]	Multivariate *p* Value
Age	72 [64–81]	74 [68–83]	**0.093**		
Sex (male)	1025 (63.4%)	78 (67.8%)	**0.020**	0.75 [0.50–1.11]	0.153
Clinical presentation					
Fever	402 (26.5%)	23 (18.7%)	**0.034**		
Pain	658 (43.4%)	46 (37.4%)	**0.030**		
Chest pain	83 (5.5%)	24 (19.5%)	**<0.001**	3.46 [1.82–6.56]	**<0.001**
Dyspnoea	162 (10.7%)	22 (17.9%)	**0.015**	1.19 [0.69–2.04]	0.520
Neurological symptoms	55 (3.6%)	12 (9.8%)	**0.002**	2.96 [1.49–5.89]	**<0.001**
Laboratory findings					
Creatinine (mg/dL)	1.94 [0.82–2.02]	2.09 [0.94–2.63]	**0.018**		
eGFR (ml/min)	54.7 [29.2–85.2]	45.2 [22.6–70.6]	**0.012**		
Urea (mg/dL)	33 [18–40]	36 [19.7–49]	**0.035**		
Glucose (mg/dL)	174 [107–210]	160 [107–184]	0.160		
HbA1c (mmol)	61 [45–73]	54 [43–62]	**<0.001**		
C-reactive protein (mg/L)	96 [19–148]	88 [16–149]	0.660		
Total cholesterol (mg/dL)	128 [101–150]	135 [104–162]	0.230		
HDL (mg/dL)	32 [24–38]	31 [21–40]	0.830		
LDL (mg/dL)	72 [50–86]	84 [49–114]	**0.070**		
Triglycerides (mg/dL)	127 [86–153]	139 [98–177]	**0.048**		
Hb (gr/dL)	11.3 [9.5–13]	11.2 [9.6–13.1]	0.430		
PLT (×10^9^/L)	306 [210–373]	249 [174–294]	**<0.001**		
PAD grade (according to Rutherford classification)					
Rutherford Grade I-3	88 (5.8%)	13 (10.6%)		*Reference*	
Rutherford Grade II-4	147 (9.7%)	24 (19.5%)		0.71 [0.32–1.71]	0.398
Rutherford Grade III-5	152 (10%)	9 (7.3%)	**<0.001**	0.44 [0.19–0.97]	**0.041**
Rutherford Grade III-6	847 (55.8%)	41 (33.3%)		0.45 [0.21–0.90]	**0.026**
Previous amputation	254 (16.7%)	11 (8.9%)	**0.007**	0.96 [0.37–2.50]	**0.012**
Presence of infections					
Bloodstream infection	119 (7.8%)	11 (8.9%)	0.12		
Lower limb infections	847 (55.8%)	41 (33.3%)	**<0.001**	0.61 [0.29–1.27]	0.332
Other infections	988 (65.1%)	55 (44.7%)	**<0.001**	0.73 [0.38–1.38]	0.336
Comorbidities					
CCI	4 [2–5]	4 [3–6]	**0.003**	1.08 [1.01–1.17]	**0.047**
CAD	626 (41.3%)	73 (59.3%)	**<0.001**		
Congestive heart failure	405 (26.7%)	42 (34.1%)	**0.017**		
Cerebrovascular disease	130 (8.6%)	19 (15.4%)	**0.021**		
Dementia	101 (6.7%)	8 (6.5%)	0.57		
COPD	179 (11.8%)	17 (13.8%)	0.29		
Diabetes	1193 (78.6%)	87 (70.7%)	**0.05**		
Chronic kidney disease	521 (34.3%)	44 (35.8%)	0.41		
Outcomes					
Length of stay (LOS)	15.73 [7.4–20.2]	18.8 [7.5–26]	**0.011**		
Death (all causes)	81 (5.3%)	46 (37.4%)	**<0.001**		

Abbreviations: HbA1c, glycated haemoglobin; HDL, high-density lipoprotein; LDL, low-density lipoprotein; Hb, haemoglobin; PLT, platelets; PAD, peripheral artery disease; CCI, Charlson Comorbidity Index; CAD, coronary artery disease; COPD, chronic obstructive pulmonary disease. Lower limb infections were excluded due to high collinearity with Rutherford’s class.

## Data Availability

The data presented in this study are available on request from the corresponding author.
